# Mesopic conditions optimise the detection of visual function loss in drivers with simulated media opacity

**DOI:** 10.1038/s41598-022-17448-w

**Published:** 2022-08-01

**Authors:** Frederick A. Asare, Roger S. Anderson, Pádraig J. Mulholland, Julie-Anne Little

**Affiliations:** 1 Centre for Optometry and Vision Science, Biomedical Sciences Research Institute, Ulster University, Coleraine, UK; 2 National Institute for Health Research (NIHR) Biomedical Research Centre at Moorfields Eye Hospital NHS Foundation Trust and UCL Institute of Ophthalmology, London, UK

**Keywords:** Health care, Medical research, Eye diseases

## Abstract

Drivers have different visual demands across varying contrast and luminance conditions. However, vision assessments for driving are typically conducted under photopic conditions. This study investigated the sensitivity of photopic and mesopic conditions to detect contrast sensitivity (CS) loss in drivers with simulated media opacities. CS was measured in forty-seven healthy drivers aged 18–50 years (mean ± SD: 25.5 ± 6.5) under photopic and mesopic-adapted luminance levels with the Pelli-Robson chart and the Mesotest II (without glare). Media opacities were simulated using white-opacity containing Lee Fog filters (1–5) and CS measured in a randomised order. A significant (*p* < 0.001) reduction in photopic CS (logCS) was measured with the Pelli-Robson chart only when media opacity was simulated with Fog filter 5 (1.53 ± 0.15, 2.8 triplets reduction) compared to baseline (1.95 ± 0.03). Mean mesopic CS demonstrated a significant (all *p* < 0.001) reduction from baseline (1.67 ± 0.14) for Fog filters 3 (1.4 triplets, 1.45 ± 0.16), 4 (2.4 triplets, 1.31 ± 0.14) and 5 (4.3 triplets, 1.02 ± 0.15). For Mesotest II, only Fog filter 5 produced a significant reduction (0.10 ± 0.09; *p* < 0.001) in mean mesopic CS from baseline (0.30 ± 0.01). Mesopic CS is more vulnerable to different levels of simulated media opacity, hence should be considered clinically when assessing visual function in older drivers at risk of media opacity.

## Introduction

High-contrast visual acuity (HCVA) remains the reference standard measure of visual function in drivers. While this is the case, it only provides information about the optimum resolution capacity of the visual system and not reflective of visual performance in complex, real-world environments where driving takes place^1,2^. On this basis, contrast sensitivity (CS) measurement has been proposed to be a better measure of central visual function in drivers, given that the driving environment contains a wide range of modulating contrasts^3^.

Despite its proposal as a better predictor of ‘real world’ functional vision than HCVA, especially in people with cataract (media opacities), it is not routinely assessed in clinical settings and, when it is, it is typically performed under photopic conditions which are not representative of the conditions in which many real-world activities occur. For instance, during driving in low-light conditions, one’s ability to detect subtle levels of contrast is what enhances the recognition of darkly-clothed pedestrians, as well as road markings and street signs^4,5^. As such, it is imperative that CS assessment is carried out under different luminance or lighting conditions to fully evaluate its impact on vision and relate this to driving performance. Given that cataract is the leading cause of visual impairment globally and it significantly affects driving especially at night, there is a need to identify the optimal functional test to detect functional vision in drivers with such condition. According to Kimlin, Black and Wood, CS under mesopic luminance (0.38 ± 0.02 cd/m^2^) is better correlated with night-time driving performance than photopic CS, hence assessment of driving performance should be considered under this luminance level^6^. Hertenstein et al. also indicate that measures of CS performed in photopic conditions cannot serve as surrogate measures in mesopic conditions^7^. These authors reported that while reduced CS measures in photopic conditions do relate closely to reduced CS in mesopic adaptation conditions, not all normal photopic CS measures correlate with normal mesopic CS. With regard to the effect of CS loss on road crashes, Owsley et al. reported that older drivers with a history of motor vehicle collision involvement are eight times more likely to have severe CS loss in their worse eye (defined as a Pelli-Robson score ≤ 1.25) than those who are crash-free^8^.

Although there are a number of different instruments for measuring CS in literature, the Pelli-Robson chart remains the most widely used as it has been found to have reasonably good repeatability under both photopic and mesopic conditions^6,9^ compared to other CS tests. The limitation to its use for mesopic assessment, however, is that at least a 5–10 min period of visual adaptation is required^9–13^. Apart from that, a standardised luminance level is also required^12^ which according to many eye care practitioners, disrupts the clinical routine of testing. For that reason, the Mesotest II (Oculus GmbH, Wetzlar Germany) has consistently been used by the German driving authority^14^ and several research groups^15–19^ to evaluate mesopic vision and fitness to drive during the hours of darkness. It assesses CS under a standardised built-in mesopic luminance of 0.032 cd/m^2^^20^.

Despite the existence of several commercially available instruments and their importance in assessing CS under different luminance or lighting conditions, they are still under-explored with regard to their abilities to detect functional loss among drivers with different levels of visual impairment, and thus have not been widely adopted in routine clinical eyecare. For instance, to our knowledge, no study has examined the ability of commercially available tests of CS to detect functional losses in drivers with media opacity under different lighting conditions. This study addresses this knowledge gap, assessing CS with commercially available instruments under photopic and mesopic luminance levels and investigating their ability to identify visual function loss from simulated media opacities.

## Methods

### Participants

Forty-seven healthy participants aged 18–50 years with no eye-disease and a full UK driving licence who met the UK vision standards for driving (i.e., binocular habitual HCVA of better than or equal to 6/12 [0.3 LogMAR] in photopic conditions) were recruited from staff and student populations at Ulster University. Inclusion criteria were that participants had no amblyopia, had not undergone cataract or refractive surgery and had no self-reported ocular pathology of the cornea, lens or retina. Ethical approval for the study was obtained from the Biomedical Sciences Ethics Filter Committee at Ulster University while written informed consent was obtained from each participant. Participation was free and voluntary, and the study adhered to the tenets of the Declaration of Helsinki.

### Sample size determination

The sample size for the study was calculated with the G*Power statistical software (version 3.1.9.4). Using a power of 80%, an effect size of 0.18 and a significance level of 5% for a one-way repeated measures ANOVA in a single group of participants with six different measurements per variable, the minimum sample size required for the study was calculated to be 35. The effect size was determined using a partial eta-squared value of 0.03 with the assumption that there will be a moderate effect of Fog filters (simulated media opacity) on participants’ baseline CS.

### Experimental set-up

#### Visual adaptation and luminance levels

Participants underwent a 10-min period of mesopic light adaptation before mesopic vision assessments were carried out which was consistent with that reported in previous studies^6,11–13,15,21^. The illumination in the study room was adjusted by using a dimmable bulb which could be regulated to produce the desired luminance. The chart luminance was then measured with a SpectroCAL MKII spectroradiometer (JETI Technische Instrumente GmbH) at three random points on the chart and then averaged to produce a mesopic chart luminance of 0.29 ± 0.02 cd/m^2^. This was found to be consistent with the mesopic luminance range (0.1–1 cd/m^2^) used in other studies^6,10,22^. Photopic CS was also measured with the Pelli-Robson CS chart with an average luminance of 118.62 ± 3.21 cd/m^2^ as measured with the SpectroCAL MKII spectroradiometer (JETI Technische Instrumente GmbH) at three random points on the chart.

#### Simulation of media opacity

**Figure 1:**
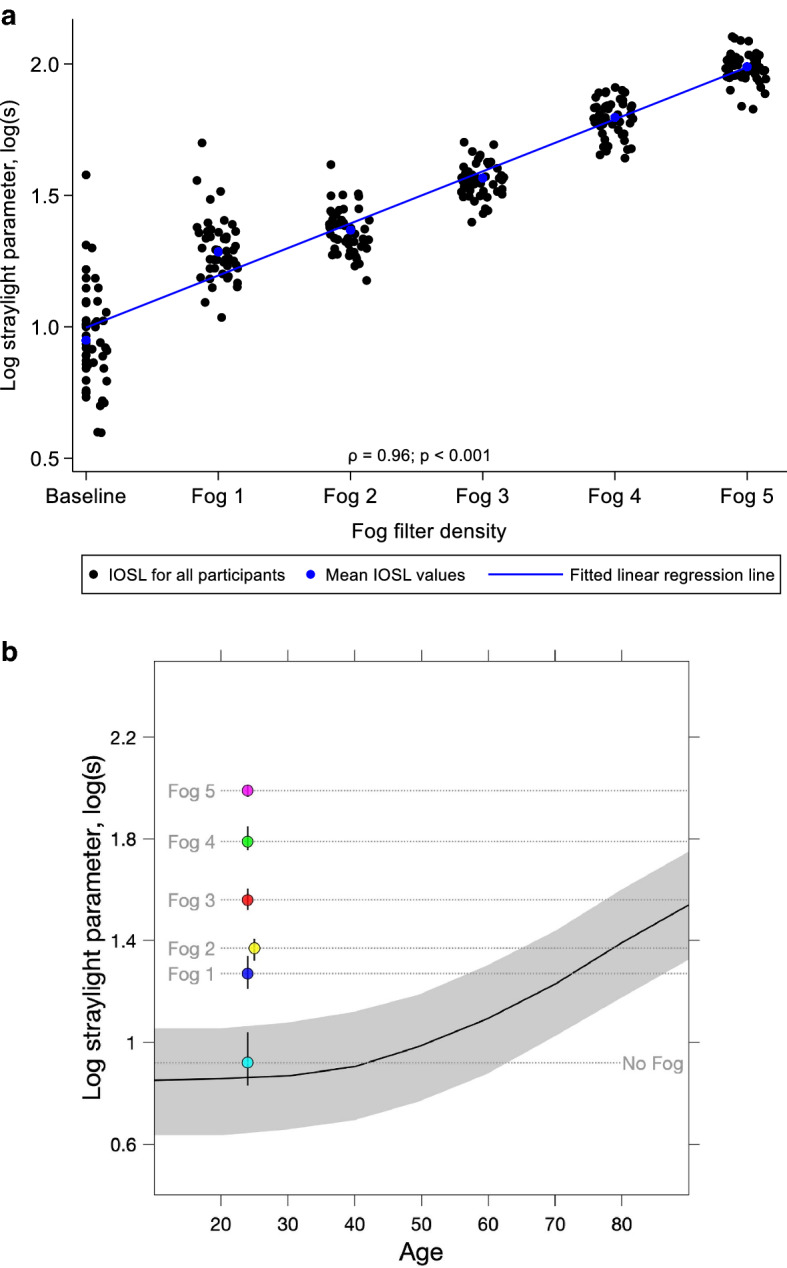
**(a)** Intraocular straylight (IOSL) values for 47 participants at baseline and under different Fog filter conditions as measured with the C-Quant straylight metre. Spearman’s correlation coefficient (ρ) shows a strong positive and a significant correlation between mean IOSL values and Fog filter density. (**b**) Effect of Fog filters on median IOSL value at baseline and under different Fog filter conditions as measured with the C-Quant straylight meter. Dark curved line = mean IOSL value for each age. Grey band = range (5–95% percentiles) of normative IOSL values for age. Coloured circles = median straylight values for all participants (with ± 1 jitter added where needed to aid visualisation) at baseline and for five Fog conditions, vertical lines = interquartile range.

Different levels of media opacity were simulated using the Lee Fog filters 1–5 (Fog filters; Lee Filters, Andover, UK) which are white opacity-containing filters which induce wide angle light scatter typical to that of intraocular scatter caused by lens opacification^23,24^. These Fog filters have mean light transmittance ranging from 0.86 (fog 1) to 0.42 (fog 5) as measured with a spectrophotometer (SpectraScan PR-650 Spectra Colorimeter; Photo Research Inc., Chatsworth, CA) directly against an achromatic CRT screen (luminance: 10 cd/m^2^; chromaticity coordinates: *x* = 0.218, *y* = 0.328)^23^. Before they were used as filters to simulate media opacity in this study, they were placed, in random sequence, in the lens holder of a C-Quant straylight meter (Oculus GmbH, Wetzlar Germany) and individual participant’s intraocular straylight (IOSL) measured with each filter. All straylight values included in the study had a reliability coefficient (Q) of ≥ 1 and an expected standard deviation (esd) of ≤ 0.08. These reliability indices were ensured by choosing the appropriate range (R) settings for each participant at each level of blur. Tests were also repeated for values outside the Q and esd estimates before they were included in the study. After analysis, it was found that Fog filters 1–3 produced mean straylight values of 1.3–1.6 log[s] which were consistent with the range of straylight values produced by early cataract (0.6–1.6 log[s]) using the C-Quant as reported in a study by De Wit et al.^25^. A Spearman’s correlation to assess the relationship between IOSL and Fog filter density also revealed that there was a strong positive correlation between the two variables which was statistically significant (ρ = 0.96; *p* < 0.001). That is, for each increase in Fog filter density, IOSL value significantly increases. Figure 1a illustrates the effect of Fog filters on participants’ IOSL measure at baseline while Fig. 1b describes how Fog filters change IOSL values of younger participants to depict IOSL values typical of ageing and significant cataract. For instance, Fog filters 1–3 take the IOSL value at a median age of 24-years to that of much older individual with age-related increase in IOSL while Fog filters 4–5 take them beyond the limits of agreement for the IOSL for the normal ageing eye into the realm of significant cataract.

#### Data collection procedure

Prior to the commencement of vision assessment, demographic data of participants which included age, gender, and self-reported presence of ocular conditions like amblyopia, cataract, cataract surgery, refractive surgery or any other ocular pathology were collected and used as inclusion/exclusion criteria. Participants’ recent refractive correction based on their clinical records from the university’s eye clinic was used for all assessments.

Participants’ HCVA was measured with the Early Treatment of Diabetic Retinopathy Study (ETDRS) chart to ensure they satisfied the VA requirement of the study. An eccentric infrared photorefractor (PlusOptix PoweRef III) was also used to measure participants’ pupil diameter under photopic conditions immediately after photopic vision assessments and then under mesopic conditions just before all mesopic CS assessments. Photopic CS was then measured with the Pelli-Robson CS chart, while mesopic CS was measured with the Pelli-Robson chart and then the Mesotest II. All measurements were finally repeated with the Fog filters 1–5 in a randomised order.

#### Photopic and mesopic CS assessment with the Pelli-Robson chart

Photopic CS was assessed binocularly for participants at baseline using the Pelli-Robson CS chart at a distance of 1 m while they wore their habitual correction, if any. The chart, which consists of 16 triplets of size 4.9 cm (2.8° at 1 m) with each optotype equivalent to a Snellen visual acuity of 6/204 at 1 m, assesses contrast sensitivity at a letter ‘characteristic’ spatial frequency of 1.00 cycle per degree (cpd). The letters in each triplet have the same contrast, with the contrast in each successive triplet decreasing by a factor of 0.15 log units^26^. During the assessment, participants were encouraged to guess letters until a full triplet was answered incorrectly. Contrast sensitivity was then scored as 0.05 log units for every correctly identified letter, with O and C being accepted interchangeably^26^. Measurements were then repeated with Fog filters 1–5 which were glazed into full aperture trial lenses and randomly placed in a trial frame immediately behind participants’ appropriate refractive correction, if any. Participants then underwent a 10-min visual adaptation after which assessment was carried out again under mesopic lighting conditions in the same manner as the photopic assessment. Mesopic CS measurements were taken at baseline after which they were repeated with Fog filters 1–5 in a randomised order.

#### Mesopic CS assessment with Mesotest II

**Figure 2:**
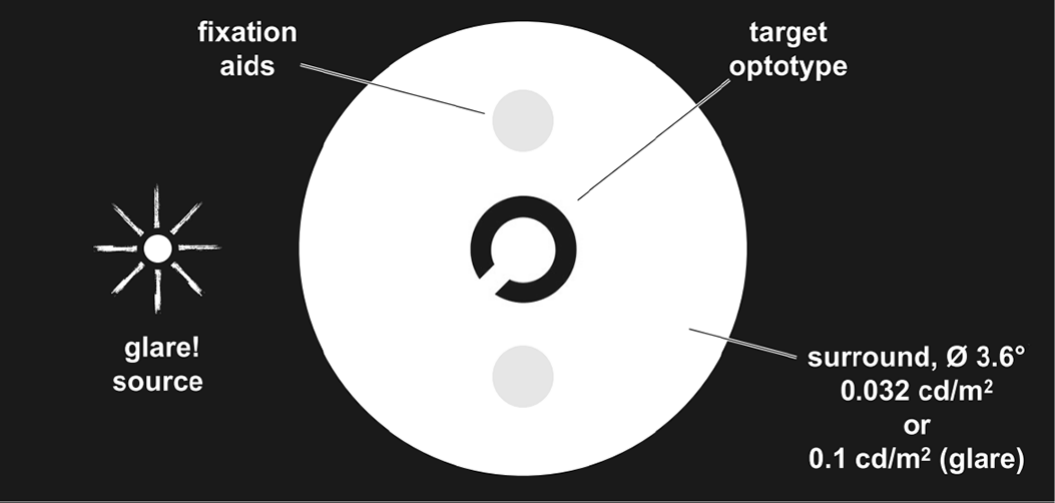
Participants’ view in the Mesotest II (Oculus GmbH, Wetzlar Germany) during testing. Adapted from instruction manual for Mesotest II (Oculus GmbH, Wetzlar Germany).

The Mesotest II is used for testing mesopic vision and glare sensitivity in the absence and presence of a glare source respectively. However, when used for CS assessment, it operates under a no glare mesopic condition which has a standardised luminance of 0.032 cd/m^2^^20^. During the assessment, participants were shown up to five Landolt Cs (equivalent to a Snellen visual acuity of 6/60 with a spatial frequency of 3 cpd) per contrast level (Table 1) and asked to identify the direction of the gap in the C (see Fig. 2) in six alternatives (up, up-right, up-left, down, down-right and down-left) starting from the highest contrast level (1:23; 0.02 logCS). The Landolt Cs are presented binocularly at 5 m. If participants correctly identified the gap in three of the five different presentations (60% correct), the next lower contrast level was tested, and testing stopped when participants failed to meet this criterion (i.e., a single reversal was achieved). The last successfully completed level determined participants’ score^7^. Participants who were unable to recognise the highest level of contrast were assigned a score 0.00 logCS (a level that cannot be achieved in the test) so that their results could be included in the analysis. Measurements were then repeated with Fog filters 1–5 which were randomly placed in a trial frame worn by participants with their appropriate refractive correction in place.

**Table 1:** Mesotest II contrast levels as proposed by manufacturers expressed in logCS.

Mesotest II contrast level (L_min_ : L_max_)	Contrast threshold Weber contrast = (L_max_ − L_min_)/L_max_	Log contrast sensitivity (logCS)
1:23	0.95	0.02
1:5	0.80	0.1
1:2.7	0.63	0.2
1:2	0.50	0.3

L_min_: Luminance for darker region, L_max_: Luminance for brighter region.

### Statistical analysis

Statistical analysis was performed using STATA 16.1 (StataCorp, College Station, TX, USA) and MATLAB (R2020B, The MathWorks Inc., USA). The data were analysed as frequency, percentages, and proportions. Mean, standard deviations, and 95% confidence intervals were also estimated for quantitative variables. A one-way repeated measures ANOVA was conducted on datasets which were approximately normally distributed and did not violate Mauchly’s test of sphericity. A pairwise comparison of means with adjustment for repeated measures was then used to assess the difference in mean between the baseline CS of participants and their CS when the different levels of media opacities were simulated under both photopic and mesopic conditions. Spearman’s correlation coefficient, LOWESS function, Passing-Bablok and Kendall’s tau correlation coefficient were then conducted to assess the level of correlation for variables that were not normally distributed while Bland–Altman analysis was conducted to assess the level of agreement between mesopic CS as measured with the Pelli-Robson CS chart and Mesotest II at baseline and under all simulated media opacity conditions. For all inferential statistical tests, an alpha of 0.05 was used to determine statistical significance.

## Results

### Characteristics of participants

A total of forty-seven licensed drivers were recruited and examined in this study. Of this number, 58% were females. The median age (IQR) of the participants was 24 years (20–31 years) and their ages ranged from 19 to 44 years while the median (IQR) years of continuous driving was 4.0 years (2.5–8.0 years).

### Photopic HCVA with the ETDRS chart

All participants had habitual binocular photopic HCVA of better than or equal to 6/6 (0.00 LogMAR) with a mean of − 0.12 ± 0.07 LogMAR (range: − 0.28 to 0.02 LogMAR). Table 2 summarises participants mean photopic HCVA (binocular) at baseline and at each level of blur.

**Table 2:** Mean SD binocular photopic HCVA at baseline and at each level of blur.

Level of blur	Mean ± SD (LogMAR)	Range (LogMAR)
Baseline	− 0.12 ± 0.07	− 0.28–0.02
Fog 1	− 0.11 ± 0.07	− 0.28–0.12
Fog 2	− 0.11 ± 0.07	− 0.28–0.12
Fog 3	− 0.10 ± 0.08	− 0.30–0.14
Fog 4	− 0.10 ± 0.07	− 0.28–0.14
Fog 5	− 0.08 ± 0.09	− 0.24–0.26

A one-way repeated measures ANOVA showed that photopic HCVA was affected by the introduction of Fog filters (F [5, 230] = 11.1; *p* < 0.001). A post-hoc analysis with the pairwise comparison of means further revealed that there was a statistically significant reduction in mean HCVA from baseline when media opacity was simulated with Fog 3 (mean difference: 0.02 ± 0.01 LogMAR; 95% CI 0.01–0.04; *p* < 0.008), 4 (mean difference: 0.02 ± 0.01 LogMAR; 95% CI 0.01–0.04; *p* = 0.001) and Fog 5 (mean difference: 0.04 ± 0.01 LogMAR; 95% CI 0.03–0.06; *p* < 0.001) after adjusting for multiple comparison using Bonferroni correction.

### Photopic and mesopic pupil diameter

The mean ± SD pupil diameter of participants as measured with the PowerRefractor was larger under mesopic condition (6.2 ± 0.7 mm; range: 4.9–7.6 mm) than photopic condition (4.7 ± 0.8 mm; range 3.2–7.0 mm) and was statistically significant (r = 0.75; *p* < 0.001).

### Photopic CS with the Pelli-Robson chart

Mean photopic CS ± SD (logCS) at baseline was 1.95 ± 0.03 (range: 1.75–1.95), and this value did not significantly change when media opacity was simulated with Fog filters 1 (1.94 ± 0.04), 2 (1.94 ± 0.04) and 3 (1.92 ± 0.10), despite significant changes in the IOSL level. However, for Fog filters 4 and 5, the mean CS ± SD was reduced to 1.82 ± 0.14 and 1.53 ± 0.15 respectively. A one-way repeated measures ANOVA showed that photopic CS was affected by the introduction of Fog filters (F [5, 230] = 219.5; *p* < 0.001). A post-hoc analysis with the pairwise comparison of means further revealed that there was a statistically significant reduction in mean CS from baseline when media opacity was simulated with Fog 4 (mean difference: 0.13 ± 0.02; 95% CI 0.07–0.19; *p* < 0.001) and Fog 5 (mean difference: 0.42 ± 0.02; 95% CI 0.36–0.47; *p* < 0.001) after adjusting for multiple comparison using Bonferroni correction.

### Mesopic CS with the Pelli-Robson chart

**Figure 3:**
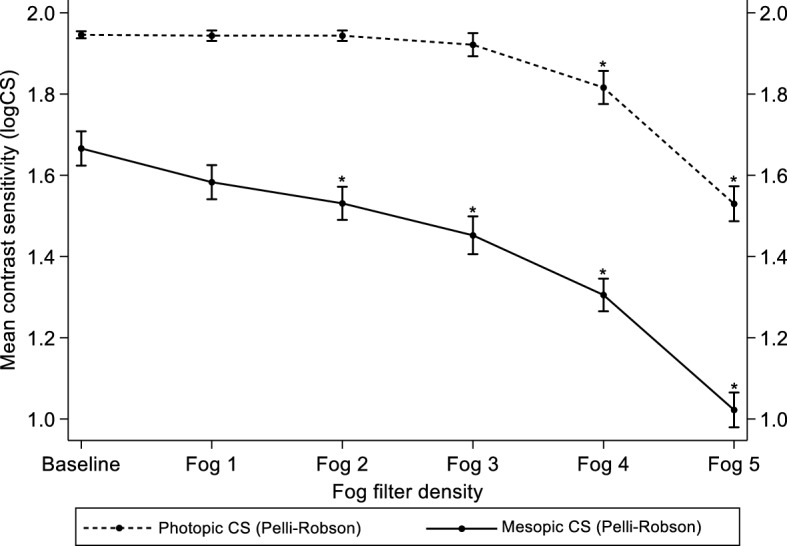
Mean photopic and mesopic CS at baseline and under different Fog filter conditions as measured with the Pelli-Robson chart. Error bars are 95% confidence interval of means. * = *p* < 0.001 and represents significant difference in CS at baseline compared with Fog filters 4 and 5 under photopic conditions and 2–5 under mesopic conditions.

The mean mesopic CS ± SD (logCS) at baseline was 1.67 ± 0.14 (range: 1.35–1.95) and decreased with the introduction of each level of simulated media opacity to a varying degree. While Fog filters 1 and 2 reduced the mean mesopic CS ± SD only slightly (1.58 ± 0.14 versus 1.53 ± 0.14), simulated media opacity with Fog filters 3, 4 and 5 reduced the mean CS ± SD to 1.45 ± 0.16, 1.31 ± 0.14 and 1.02 ± 0.15 respectively. A one-way repeated measures ANOVA indicated that mesopic CS was affected by the introduction of Fog filters (F [5, 230] = 534.2; *p* < 0.001). A post-hoc analysis with the pairwise comparison of means, however, revealed that there was a statistically significant reduction in mean CS from baseline when media opacity was simulated with Fog filters 2–5 (all *p* < 0.001) after adjusting for multiple comparison using Bonferroni correction. Figure 3 describes the effect of Fog filters on participants’ mean photopic and mesopic CS as measured with the Pelli-Robson chart.

### Relationship between photopic and mesopic CS measured with Pelli-Robson chart

**Figure 4:**
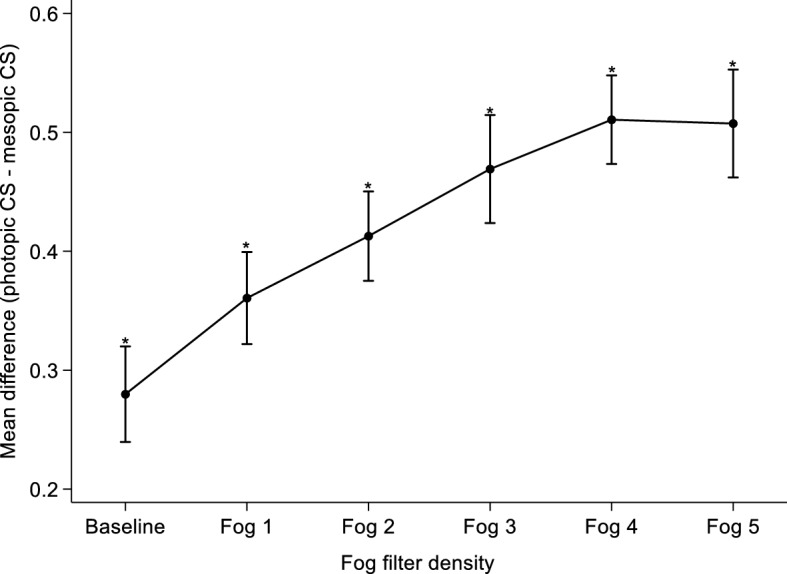
Mean difference between photopic and mesopic CS at baseline and under different Fog filter conditions as measured with the Pelli-Robson chart. Error bars are 95% confidence interval of the mean difference. * = *p* < 0.05 representing significant difference between mean photopic and mesopic CS at baseline and when media opacity was simulated with all Fog filters.

Mesopic CS was lower at baseline and proportionally reduced to a greater extent when media opacity was simulated with all Fog filters (1–5) compared to photopic CS. At baseline, mean CS measured under mesopic conditions was significantly reduced by nearly two triplets (0.28 logCS) compared to photopic conditions, while with Fog 1 it was reduced by 2.4 triplets (0.36 logCS). Fog filters 2 and 3 reduced mean mesopic CS by 2.7 (0.41 logCS) and 3.1 triplets (0.47 logCS) respectively compared to when measured under photopic conditions. Simulated media opacity with Fog 4 and 5 reduced mesopic CS by the same amount (i.e. 3.4 triplets (0.51 logCS)) compared to measures under photopic conditions. Figure 4 describes the mean difference between photopic and mesopic CS at baseline and at different levels of media opacity.

### Mesopic CS with the Mesotest II

**Figure 5:**
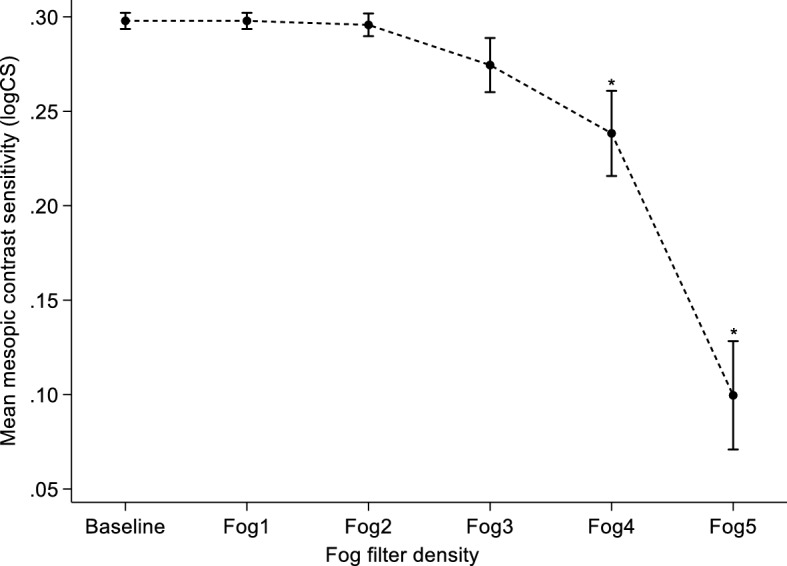
Mean mesopic CS at baseline and under different Fog filter conditions as measured with the Mesotest II. Error bars are 95% confidence interval of means. * = *p* < 0.001 and represents significant difference in CS at baseline compared with Fog filters 4 and 5.

The mean mesopic CS ± SD (logCS) at baseline was 0.30 ± 0.01 (range: 0.2–0.3) and was unaffected by media opacity simulated with Fog filters 1 (0.30 ± 0.01), 2 (0.30 ± 0.02) and 3 (0.27 ± 0.05). However, for simulation with Fog filters 4 and 5, mean mesopic CS ± SD was reduced from baseline to 0.24 ± 0.08 and 0.10 ± 0.09 respectively. A one-way repeated measures ANOVA revealed that mesopic CS as measured with the Mesotest II was affected by the introduction of Fog filters (F [5, 230] = 121.4; *p* < 0.001). A post-hoc analysis with the pairwise comparison of means further indicated that there was a statistically significant reduction in mean CS from baseline to when media opacity was simulated with Fog filters 4 (mean difference: 0.06 ± 0.01; 95% CI 0.03–0.09; *p* < 0.001) and 5 (mean difference: 0.20 ± 0.01; 95% CI 0.16–0.23; *p* < 0.001) after adjusting for multiple comparison using Bonferroni correction. Figure 5 describes the effect of Fog filters on participants’ mean mesopic CS as measured with the Mesotest II.

### Correlation between mesopic CS measured with Pelli-Robson chart and Mesotest II

**Figure 6:**
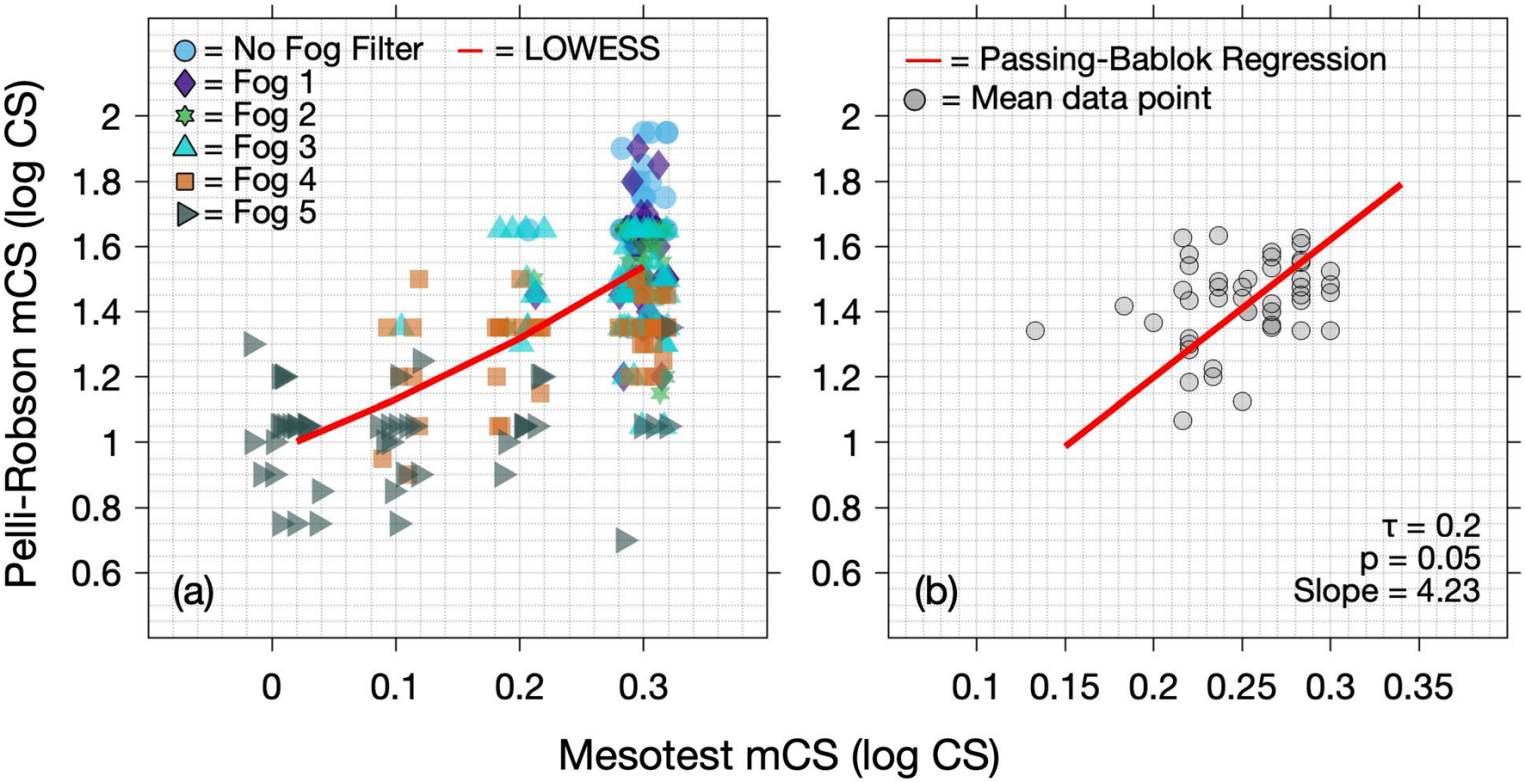
(**a**) LOWESS function (red line) to assess the relationship between mesopic CS as measured with the Pelli-Robson chart and Mesotest II at baseline and with all Fog filters; (**b**) a scatter plot of mean CS values across all Fog filter conditions for each participant with Passing–Bablok regression (red line) and Kendall’s tau correlation coefficient (Ʈ).

Mesopic CS measures from the two instruments were analysed to assess the strength of correlation between them using a LOWESS function which qualitatively showed a positive linear trend between mesopic CS as measured with the Pelli-Robson and the Mesotest II (Fig. 6a). A Passing-Bablok regression and Kendall’s tau correlation coefficient (Ʈ) were further conducted over the mean CS values across all Fog filter conditions for each participant and this revealed a weak correlation (Ʈ = 0.2, *p* = 0.05) between mesopic CS values measured with the Pelli-Robson chart and the Mesotest II (Fig. 6b).

### Agreement between mesopic CS measured with Pelli-Robson chart and Mesotest II

**Figure 7:**
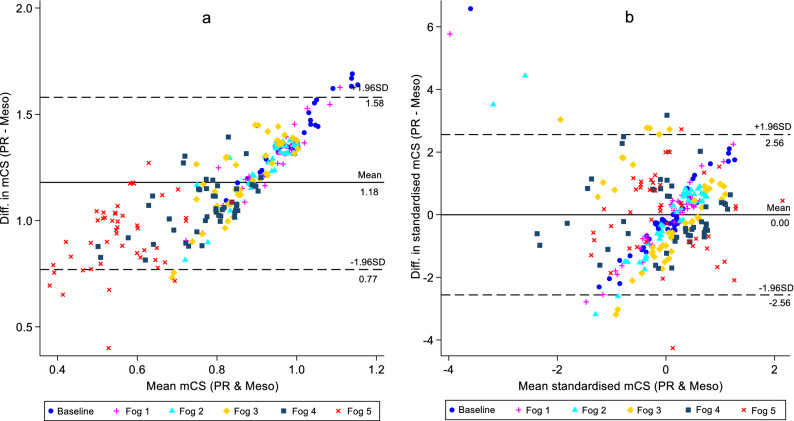
Bland–Altman plot of raw (**a**) and standardised (**b**) mesopic contrast sensitivity (mCS) measures between Pelli-Robson chart (PR) and Mesotest II (Meso) at baseline and with all Fog filters. Solid lines represent the mean difference in mesopic CS between the two instruments while dashed lines represent the 95% upper and lower limits of agreement.

The two instruments were assessed for level of agreement between them using Bland–Altman analysis with the raw (unstandardised) and standardised measures using z-scores (Fig. 7a,b). The analysis revealed that the mean difference ± SD (logCS) between the raw (unstandardised) mesopic CS measures with the Pelli-Robson CS chart and Mesotest II was 1.18 ± 0.21, while the lower and upper limits of agreement were 0.77 and 1.58 respectively (Fig. 7b). For standardised measures, it was revealed that the mean difference ± SD between the mesopic CS as measured with the Pelli-Robson CS chart and Mesotest II was − 0.00 ± 1.31, with the lower and upper limits of agreement being − 2.56 and 2.56 respectively (Fig. 7b).

## Discussion

The results show that there was no clinically significant effect of photopic condition on detection of vision loss in drivers with simulated media opacity. While Fog filters 1–4 resulted in a single-letter reduction (0.02 LogMAR) in photopic HCVA from baseline, Fog 5 demonstrated only a two-letter reduction (0.04 LogMAR) from baseline VA. This was consistent with results from a study by Castro-Torres et al.^27^ which showed no difference between participants’ mean baseline photopic HCVA and when Hoya filters (Fog A, B & A + B) and B + W Fog_1 filter were used. This trend was also in line with another study by Martino et al.^28^ in which they assessed VA binocularly wearing different Fog filters in the dominant eye and reported that on average, VA reduced by only one line.

Mesopic conditions, on the other hand, were found to be particularly essential and more sensitive to reveal visual function loss (reduction in CS) among drivers with varying degrees of media opacity than photopic conditions. Primarily, CS measured with the Pelli-Robson CS chart under mesopic condition produced 1.4, 2.4 and 4.3 triplets reduction with Fog 3, 4 and 5 respectively from baseline while under photopic condition, only Fog 5 demonstrated nearly three triplets reduction in CS from baseline. Under mesopic condition, the Mesotest II demonstrated a two-contrast level (0.20 logCS) reduction in CS from baseline with Fog filter 5. Considering the vision requirements for driving at night by the German driving authority using the Mesotest II, drivers with such level of cataract (media opacity simulated with Fog 5) would be excluded from operating public transports as the minimum mesopic CS for operating such vehicles is 0.20 logCS which is a line reduction from the expected CS value of 0.30 logCS^14^ and not two-line reduction as observed in this study. The floor effect observed with the Fog filter 5 on mesopic CS when measured with the Mesotest II could be explained by the fact that the Mesotest II has a comparatively smaller dynamic range of contrast values (0.02 logCS to 0.3 logCS), has non-uniform scale due to the unequal difference between the first and second contrast levels and has a very low difference of 0.10 logCS between the remaining two levels. Due to this, some studies suggest that it has poor repeatability, hence, its usage for predicting driving performance might result in falsely labelling a driver as fit or unfit to drive in mesopic driving conditions^7,16^.

With regard to the strength of correlation between mesopic CS as measured with the Pelli-Robson CS chart and the Mesotest II, it was observed that there was a positive linear correlation between them (Fig. 6a). Although a larger proportion of the mesopic CS values were recorded at the lowest contrast level (that is, highest CS of 0.3 logCS) of the Mesotest II resulting in a floor effect, these measurements did not necessarily suggest a higher CS measure with the Pelli-Robson CS chart especially in the presence of Fog filters 3–5. For instance, a higher CS measure with the Mesotest II corresponded to either a lower or higher CS measure with the Pelli-Robson CS chart (Fig. 6a). However, for lower mesopic CS values with the Mesotest II, there was corresponding lower measures with the Pelli-Robson CS chart. This was further confirmed in Fig. 6b which plotted mean CS values across all Fog filters for each participant using the Passing-Bablok and Kendall’s tau correlation coefficient which revealed a weak correlation between mesopic CS measures from the two instruments.

For the agreement between the two instruments (Mesotest II and Pelli-Robson chart), Bland–Altman analysis with raw (unstandardised) mesopic CS measures (Fig. 7a) showed that there was a systematic difference between the measurements as the line of equality did not fall within the confidence interval of the mean difference (95% CI 1.15–1.20 logCS). There was also a proportional bias which suggested that even though the two instruments measure the same parameter (mesopic CS), they do not agree with each other, hence cannot be considered appropriate when used interchangeably. Measurements were further standardised using z-scores (Fig. 7b) in order to account for the varying range of contrast values between the two instruments and analysis re-conducted. This resulted in a mean difference of zero between the two instruments which was accounted for by the standardisation. This eliminated the proportional bias, but still revealed large 95% limits of agreement beyond what would be a sufficiently sensitive measure of change/difference for CS. Given that a reduction of more than 0.3 logCS (2 triplets or 6 letters) on the Pelli-Robson chart has an impact on task performance and quality of life^29,30^, the mean of the difference observed between the two instruments (> 0.3 logCS) suggests their incomparability. Overall, the Mesotest II was poor at discriminating between different levels of media opacity simulated with Fog filters, relative to the Pelli-Robson measure, thus limiting the effectiveness of this instrument for detection of CS loss.

Comparing CS measures under photopic and mesopic conditions at baseline and when media opacities were simulated, it was found that mesopic conditions significantly affected measurements at all levels. Even at baseline, participants’ mean CS as measured with the Pelli-Robson CS chart was reduced by two triplets when measured under mesopic luminance compared to photopic luminance. This highlights the importance of assessing drivers under mesopic condition to be able to adequately inform them about their night-time driving performance. The reduction in CS at baseline under mesopic compared to photopic luminance could be due to the fact that under mesopic luminance, pupil size increases (mesopic pupil size: 6.22 ± 0.70 mm vrs photopic pupil size: 4.72 ± 0.82 mm as observed in this study) which allows greater amount of light into the eyes and a subsequent increase in aberrations^31^. On the other hand, higher reduction in CS in mesopic than photopic conditions with all Fog filters could be explained on the basis that Fog filters simulate media opacities (cataract) which increase the amount of light scatter in the eyes, cause a veiling luminance over participants’ retinal image and result in CS reduction^32,33^. Moreover, it has been found that the Weber fraction for photopic luminance (0.015) is lower than that of mesopic and scotopic luminance (0.14). Thus, under mesopic luminance, individuals tend to have comparatively higher thresholds, hence lower CS as compared to photopic luminance. Under mesopic conditions, receptive fields get enlarged which reduce spatial resolution and thus CS when measured using a recognition task^34^.

It is evident that CS assessment under mesopic condition is key in drivers with and at risk of media opacities. For instance, according to Owsley et al.^35^, cataract, which results in CS reduction, exacerbates vision-related night driving difficulties in older adults who drive^36,37^. As such, assessment of mesopic CS among older drivers should be included as a standard clinical test. This was further confirmed in a study by Kimlin, Black and Wood which reported that CS under mesopic luminance is better correlated with night-time driving performance than photopic CS^6^. A recent study has also revealed that photopic CS testing may not provide enough understanding of future crash risk at the older-driver population level, rather, mesopic vision tests may be a more comprehensive assessment of the visual system’s ability to process the roadway environment^38^.

While the Pelli-Robson CS chart is able to reveal different levels of visual function loss as seen from its mesopic CS assessment, its use in clinical practice for mesopic assessment is hampered by factors including visual adaptation period and a standardised mesopic luminance. Nonetheless, a number of studies, including this study, have employed a 10-min visual adaptation period and a mesopic luminance range of 0.1–1 cd/m^2^ to explore the impact of mesopic conditions on visual function assessment^6,10–13,21,22^. While creation of mesopic conditions varies between studies, the mesopic luminance level for this study was 0.29 ± 0.02 cd/m^2^ which was achieved by adjusting illumination of a dimmable bulb in the study room and this could be employed by eye care practitioners.

Importantly, the use of a sample of healthy normal vision drivers with no significant ocular pathologies provided investigators in this study with the opportunity to simulate the same levels of media opacities without the confounding factors of other age-related co-morbidities. Each participant in effect acted as their own control. Further, simulation of media opacity with the Lee Fog filters were found to be appropriate as they significantly increased IOSL in participants, thus producing a greater amount of light scatter typical of intraocular scatter produced by true lens opacification. For instance, Fog filters 1–3 produced mean straylight values between 1.30–1.60 log[s] which were consistent with the amount of light scatter (i.e.: straylight values) produced by early cataract (0.6–1.6 log[s]) as reported by De Wit et al.^25^. In terms of types of filters, straylight values recorded in this study using the LEE Fog filters (1–3) were found to be in conformity with monocular straylight values obtained using Bangerter foils (BF_0.2 to BF_0.6; 1.38–1.64 log[s]), Hoya Fog B (1.26 log[s]), Fog A + B (1.48 log[s]) and B + W Fog 1 (1.63 log[s]) in a study by Castro-Torres et al.^27^, even though they were used to simulate general retinal image quality degradation and not cataract per se. According to De Wit et al.^25^, the B + W Fog filters 1–3 and the Hoya Fog A & B are not appropriate for simulation of early cataract. It is also worth pointing out that straylight values from the current study, using LEE Fog filters (1–3), were somewhat higher than those recorded with Tiffen Black Pro-Mist 2 filters (1.12 log[s]^25^; 1.13 log[s]^27^), Bangerter foils_0.8 (1.15 log[s]) or Hoya Fog A (1.22 log[s])^27^ which have previously been used as early-cataract-simulating filters.

Notably, the use of the Pelli-Robson chart and the Mesotest II also restricted the assessment of CS to two levels of spatial frequencies (1 cpd vs. 3 cpd) which limits the ability to effectively plot a CS function using the data from this study as for a complete evaluation of CS, a wider range of spatial frequencies is needed. This could be investigated in future studies.

## Conclusion

Mesopic CS is more vulnerable to different levels of simulated media opacity, hence should be considered clinically when assessing visual function in drivers who are at risk of media opacity to be able to adequately furnish them with the appropriate advice on the driving behaviours. However, since the optimum mesopic testing luminance under which CS should be assessed is still under-explored, future studies could be directed at developing standardised mesopic luminance level for testing as well as appropriate visual adaptation period before assessment commences. In addition, given that CS is crucial in driving, an appropriate evidence-based CS cut-off values considered safe and unsafe for driving should further be investigated.

## Data Availability

The datasets generated during and/or analysed during the current study are available from the corresponding author on reasonable request.

## References

[1] Haegerstrom-Portnoy G, Schneck ME, Brabyn JA (1999). Seeing into old age: Vision function beyond acuity. Optom. Vis. Sci..

[2] Woods RL, Wood JM (1995). The role of contrast sensitivity charts and contrast letter charts in clinical practice. Clin Exp Optom..

[3] Wood JM (2019). Driving toward a new vision: Understanding the role of vision in driving. Optom. Vis. Sci..

[4] Owens DA, Wood JM, Owens JM (2007). Effects of age and illumination on night driving: A road test. Hum. Factors.

[5] Wood JM, Owens DA (2005). Standard measures of visual acuity do not predict drivers’ recognition performance under day or night conditions. Optom. Vis. Sci..

[6] Kimlin JA, Black AA, Wood JM (2017). Nighttime driving in older adults: effects of glare and association with mesopic visual function. Investig. Ophthalmol. Vis. Sci..

[7] Hertenstein H, Bach M, Gross NJ, Beisse F (2016). Marked dissociation of photopic and mesopic contrast sensitivity even in normal observers. Graefe’s Arch. Clin. Exp. Ophthalmol..

[8] Owsley C, Stalvey BT, Wells J, Sloane ME, McGwin G (2001). Visual risk factors for crash involvement in older drivers with cataract. Arch. Ophthalmol..

[9] Puell MC, Palomo C, Sánchez-Ramos C, Villena C (2004). Normal values for photopic and mesopic letter contrast sensitivity. J. Refract. Surg..

[10] Puell MC (2012). Impaired mesopic visual acuity in eyes with early age-related macular degeneration. Investig. Ophthalmol. Vis. Sci..

[11] Koefoed VF, Baste V, Roumes C, Høvding G (2015). Contrast sensitivity measured by two different test methods in healthy, young adults with normal visual acuity. Acta Ophthalmol..

[12] Lin RJ, Ng JS, Nguyen AL (2015). Determinants and standardization of mesopic visual acuity. Optom. Vis. Sci..

[13] Haughom B, Strand TE (2013). Sine wave mesopic contrast sensitivity: Defining the normal range in a young population. Acta Ophthalmol..

[14] Ophthalmological G.D.O. [German, Society]. Driving fitness assessment for road traffic (2008).

[15] Puell MC (2004). Mesopic contrast sensitivity in the presence or absence of glare in a large driver population. Arch. Clin. Exp. Ophthalmol..

[16] Van Rijn LJ (2005). Measurement of stray light and glare: comparison of nyktotest, mesotest, stray light meter, and computer implemented stray light meter. Br. J. Ophthalmol..

[17] Wilhelm H (2013). Assessment of mesopic and contrast vision for driving licences: which cut-off values, which methods are appropriate?. Klin. Monbl. Augenheilkd..

[18] Urwyler P (2015). Age-dependent visual exploration during simulated day and night driving on a motorway: A cross-sectional study. BMC Geriatr..

[19] Uthoff D, Hebestedt K, Duncker G, Sickenberger H (2013). Multicentric study regarding assessment of the driving ability of LASIK and orthokeratology patients compared with conventionally corrected persons. Klin. Monbl. Augenheilkd..

[20] van den Berg TJTP (2009). Disability glare in the aging eye. Assessment and impact on driving. J. Optom..

[21] Hiraoka T, Hoshi S, Okamoto Y, Okamoto F, Oshika T (2015). Mesopic functional visual acuity in normal subjects. PLoS ONE.

[22] Pesudovs K, Marsack JD, Donnelly WJ, Thibos LN, Applegate RA (2004). Measuring visual acuity: Mesopic or photopic conditions, and high or low contrast letters?. J. Refract. Surg..

[23] Anderson RS, Redmond T, Rodney MD, Breslin KMM, Zlatkova MB (2009). The robustness of various forms of perimetry to different levels of induced intraocular stray light. Investig. Ophthalmol. Vis. Sci..

[24] Zlatkova MB, Coulter EE, Anderson RS (2006). The effect of simulated lens yellowing and opacification on blue-on-yellow acuity and contrast sensitivity. Vis. Res..

[25] De Wit GC, Franssen L, Coppens JE, Van Den Berg TJTP (2006). Simulating the straylight effects of cataracts. J. Cataract Refract. Surg..

[26] Elliott DB, Bullimore MA, Bailey IL (1991). Improving the reliability of Pelli-Robson contrast sensitivity test. Clin. Vis. Sci..

[27] Castro-Torres JJ, Martino F, Casares-López M, Ortiz-Peregrina S, Ortiz C (2021). Visual performance after the deterioration of retinal image quality: Induced forward scattering using Bangerter foils and fog filters. Biomed. Opt. Express.

[28] Martino F (2022). Effect of interocular differences on binocular visual performance after inducing forward scattering. Ophthalmic Physiol. Opt..

[29] West SK (2002). How does visual impairment affect performance on tasks of everyday life? The SEE project. Arch. Ophthalmol..

[30] Rubin GS (2001). The association of multiple visual impairments with self-reported visual disability: SEE project. Investig. Ophthalmol. Vis. Sci..

[31] Strang NC, Atchison DA, Woods RL (2008). Effects of defocus and pupil size on human contrast sensitivity. Ophthalmic Physiol. Opt..

[32] Guber I (2011). Reproducibility of straylight measurement by C-Quant for assessment of retinal straylight using the compensation comparison method. Graefe’s Arch. Clin. Exp. Ophthalmol..

[33] Van den Berg TJTP, Franssen L, Kruijt B, Coppens JE (2013). History of ocular straylight measurement: A review. Z. Med. Phys..

[34] Morland A, Molz B, Lowndes R, Gouws A, Baseler H (2018). Population receptive fields in V1 enlarge as luminance is reduced from photopic to scotopic levels. J. Vis..

[35] Owsley C, Stalvey B, Wells J, Sloane ME (1999). Older drivers and cataract: Driving habits and crash risk. J. Gerontol A. Biol. Sci. Med. Sci..

[36] Naumann RB, Dellinger AM, Kresnow M (2011). Driving self-restriction in high-risk conditions: How do older drivers compare to others?. J. Saf. Res..

[37] Gilhotra JS, Mitchell P, Ivers R, Cumming RG (2001). Impaired vision and other factors associated with driving cessation in the elderly: The blue mountains eye study. Clin. Exp. Ophthalmol..

[38] Owsley C, Swain T, Liu R, McGwin G, Kwon MY (2020). Association of photopic and mesopic contrast sensitivity in older drivers with risk of motor vehicle collision using naturalistic driving data. BMC Ophthalmol..

